# Surface Immobilization Chemistry of a Laminin-Derived Peptide Affects Keratinocyte Activity

**DOI:** 10.3390/coatings10060560

**Published:** 2020-06-11

**Authors:** Nicholas G. Fischer, Jiahe He, Conrado Aparicio

**Affiliations:** Minnesota Dental Research Center for Biomaterials and Biomechanics, University of Minnesota, 515 Delaware Street S.E., Minneapolis, MN 55455, USA

**Keywords:** surfaces, peptides, immobilization, keratinocytes, hemidesmosomes, dental implants, peri-implantitis, laminin

## Abstract

Many chemical routes have been proposed to immobilize peptides on biomedical device surfaces, and in particular, on dental implants to prevent peri-implantitis. While a number of factors affect peptide immobilization quality, an easily controllable factor is the chemistry used to immobilize peptides. These factors affect peptide chemoselectivity, orientation, etc., and ultimately control biological activity. Using many different physical and chemical routes for peptide coatings, previous research has intensely focused on immobilizing antimicrobial elements on dental implants to reduce infection rates. Alternatively, our strategy here is different and focused on promoting formation of a long-lasting biological seal between the soft tissue and the implant surface through transmembrane, cell adhesion structures called hemidesmosomes. For that purpose, we used a laminin-derived call adhesion peptide. However, the effect of different immobilization chemistries on cell adhesion peptide activity is vastly unexplored but likely critical. Here, we compared the physiochemical properties and biological responses of a hemidesmosome promoting peptide immobilized using silanization and copper-free click chemistry as a model system for cell adhesion peptides. Successful immobilization was confirmed with water contact angle and X-ray photoelectron spectroscopy. Peptide coatings were retained through 73 days of incubation in artificial saliva. Interestingly, the non-chemoselective immobilization route, silanization, resulted in significantly higher proliferation and hemidesmosome formation in oral keratinocytes compared to chemoselective click chemistry. Our results highlight that the most effective immobilization chemistry for optimal peptide activity is dependent on the specific system (substrate/peptide/cell/biological activity) under study. Overall, a better understanding of the effects immobilization chemistries have on cell adhesion peptide activity may lead to more efficacious coatings for biomedical devices.

## 1. Introduction

A repertoire of chemical routes exists for immobilizing biomolecules on biomedical device surfaces. Peptide immobilization in particular has gained widespread investigation due to peptides’ functional diversity, ease of manufacture and derivation, and defined structures [1,2]. Given this popularity, a number of different peptide immobilization chemistries have been used to anchor peptides to a material surface [3,4]. Such peptide immobilization chemistries have been exploited to create a number of exciting peptide-based strategies for surface engineering of biomedical surfaces (among others): cell adhesion surfaces [5,6] using so called “cell adhesion peptides” [7,8], antimicrobial surfaces [9,10], combined antimicrobial and cell adhesion surfaces [11–13], biosensors [14], selective attachment of cells [15,16], tri-biomolecule surfaces [17], antifouling surfaces [18], and biomineralization [19,20].

A vast number of factors affect peptide immobilization quality such as environmental pH, surface topography, surface charge, surface polarity, peptide–peptide interactions, and immobilization chemistry [21,22]. These factors affect surface density, orientation/domain exposure, peptide intra- and intermolecular structure, and overall biological activity. One easily controllable factor in these systems is the immobilization chemistry [23]. In general, immobilization chemistries are divided into two classes based on whether they are chemoselective or not. Chemoselectivity is potentially important for peptide biological activity as a means to precisely control conformation vis-à-vis activity [24]. Silanization, a process which immobilizes an organofunctional alkoxysilane molecule via hydroxyl groups at the surface of the material which then reacts with peptides, is typically not chemoselective but is attractive because of its long history in biomaterials and relative ease of use [25,26]. On the other hand, strain-promoted, copper-free azide–alkyne click chemistry immobilizes azide or dibenzocyclooctyne (DIBO)-bearing peptides with chemoselectivity but requires additional expensive peptide synthesis steps [27,28]. Little attention has been paid to the comparison of biological outcomes from cell adhesion peptides immobilized with different chemistries despite decades of work immobilizing peptides on surfaces: this is in stark contrast to molecules other than peptides, like poly(ethylene) glycol (PEG) immobilization [29]. However, previous work has shown significant differences in antimicrobial activity between antimicrobial peptides immobilized with surface-initiated atom transfer radical polymerization (SI-ATRP) versus silanization [30].

**Figure 1.**
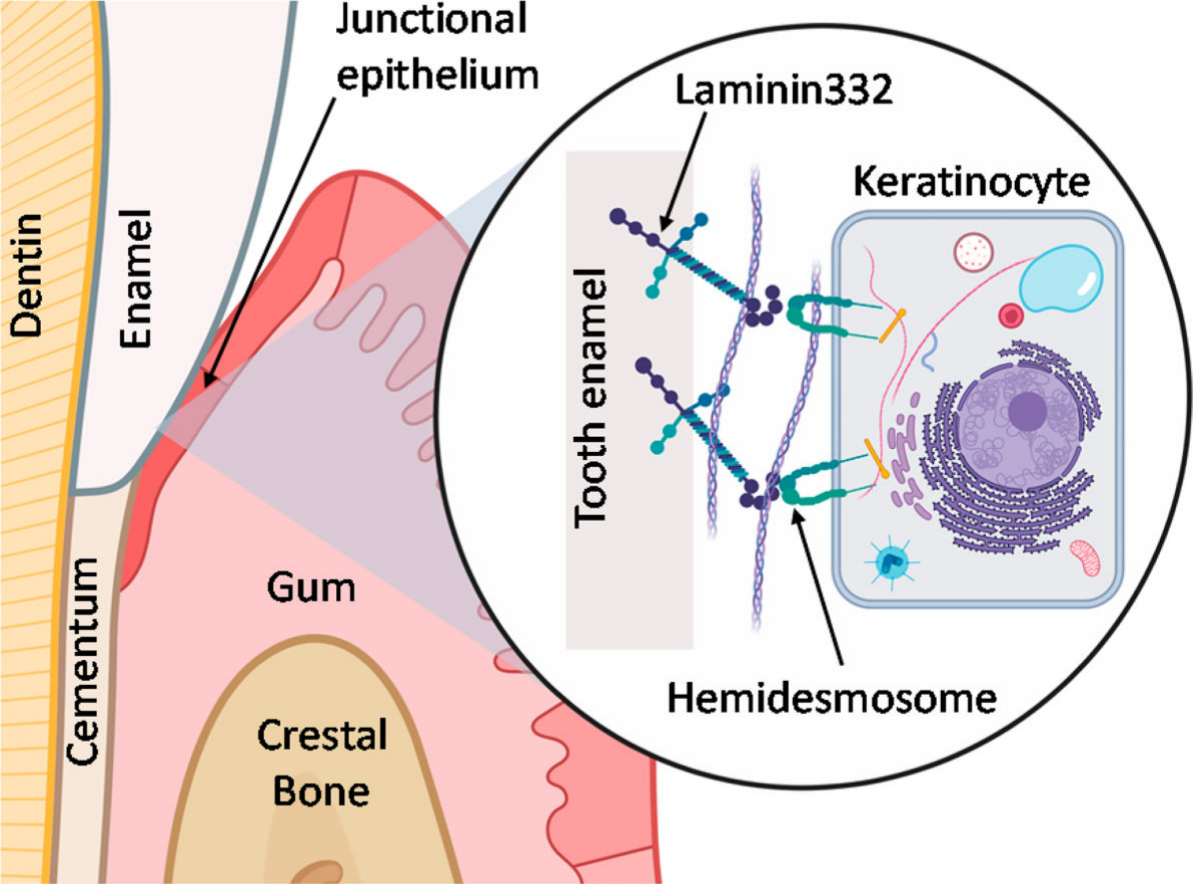
Schematic of hemidesmosome formation by keratinocytes at the junctional epithelium of the oral mucosa (gum) to attach to the tooth.

Here, we aim to compare the physical and chemical properties and biological responses of a cell adhesion peptide immobilized using both silanization and click chemistry. We selected peri-implantitis, or dental implant infection, as a model disease in need of biomedical device innovations. Peri-implantitis rates vary based on many factors but a recent meta-analysis reports an implant-based prevalence of 9.25% and a patient-based prevalence of 19.83% [31]. Other biomedical devices suffer a similar fate [32]; implantable auditory assist devices demonstrate a 26% failure rate [33] and infections occur in up to 77% of individuals with bone-anchored orthopaedic devices [34]. We have selected the peptide LamLG3 identified [35] from a globular module (LG3) of laminin332 (LM332) capable of inducing hemidesmosome (HD) formation from oral keratinocytes in the soft tissue surrounding dental implants [5,36,37]. HDs are a transmembrane “link” or “rivet” between teeth and gingiva (Figure 1), as the gingiva forms a protective, physical barrier for the tooth, or dental implant, against biofilm invasion [38,39]. Enhanced HD formation on dental implants may contribute to enhanced soft tissue healing around implants and improve their longevity.

Expected effects of click chemistry’s chemoselectivity immobilization of the LamLG3 peptides are homogeneous structural display of the tethered peptides on the surface and less restricted peptide interactions with cells than non-selective silanization. Thus, we hypothesized that coatings of LamLG3 immobilized via click chemistry (D-LamLG3) upregulate hemidesmosome formation more in comparison to coatings of LamLG3 immobilized via silanization (S-LamLG3). Overall, a better understanding of immobilization chemistry effects on cell adhesion peptide activity may lead to more efficacious coatings for biomedical devices.

## 2. Materials and Methods

### 2.1. Surface Synthesis

LamLG3-modified peptides were produced by solid-phase peptide synthesis (AAPPTec, Louisville, KY, USA). The N-terminus of each LamLG3 domain was modified with three glycines, as a flexible spacer block, plus an azide-conjugated lysine (KN3-LamLG3, K_N3_-GGG-PPFLMLLKGSTRFC, >95% purity) for click chemistry immobilization or two lysines (2K-LamLG3, KK-GGG-PPFLMLLKGSTRFC, >98% purity) to promote surface immobilization via silanization [21]. Mass spectrometry and high performance liquid chromatography of synthesized peptides is provided in Figures S1 and S2. Borosilicate glass disks (Yuanbo Engineering Co., Ltd., Hengshui, China) were activated by plasma cleaning (O_2_ for 20 min; PDC-32G, Harrick Plasma, Ithaca, USA) to expose reactive hydroxyl moieties. Plasma-activated disks (pGlass) were silanized as previously described by us [6]. Briefly, disks were placed under an N_2_-rich atmosphere and immersed in pentane containing 0.05 M *N,N*-diisopropylethylamine and 0.5 M (3-chloropropyl)-triethoxysilane (all obtained from Sigma-Aldrich, St. Louis, MO, USA) overnight and rinsed. Silanized, plasma-activated glass (pGlass-sil) were then immersed in 0.1 mM dibenzocyclooctyne-amine (DIBO, Sigma-Aldrich) in 0.1 M Na_2_CO_3_ buffer overnight and cleaned with solvents (pGlass-DIBO). These disks were then immersed in 0.1 mM KN3-LamLG3 solution in 0.1 M Na_2_CO_3_ buffer (pH = 9.5) overnight and again cleaned with solvents yielding D-LamLG3 surfaces. (It should be noted that this reaction is pH independent from pH = 2–12 [40].) A complete discussion of the reaction mechanism for this type of click chemistry is reviewed elsewhere [41]. Alternatively, disks were immersed in 0.1 mM 2K-LamLG3 solution immediately after reacting in pentane and rinsing to yield S-LamLG3 surfaces (i.e., disks).

### 2.2. Surface Characterization

#### 2.2.1. X-ray Photoelectron Spectroscopy (XPS)

An X-ray photoelectron spectroscopy (XPS) spectrometer (PHI 5000 VersaProbe III, ULVAC Inc., Chigasaki, Japan) was used to assess the surface elemental composition with a monochromatic Al Kα X-ray source (45°, 50 W, 1486.6 eV, sampling area; 200 μm diameter spot) of the peptide-immobilized surfaces and controls. Survey spectra were collected with a step size of 1.0 eV and a pass energy of 280.0 eV with charge compensation. Spectra were calibrated to the C 1*s* signal at 284.8 eV using the associated software (MultiPak, 9.6.0).

#### 2.2.2. Water Contact Angle (WCA)

Water contact angles (WCAs) were obtained using the sessile-drop method (>18 MΩ deionized water; 2 μL droplet) to assess surface wettability using a contact angle goniometer (DM-CE1, Kyowa, Niiza, Japan). Dynamic contact angles were also determined for 60 s every 1 s. Three disks per group were analyzed.

#### 2.2.3. Coating Durability

D-LamLG3 and S-LamLG3 coatings were incubated (37 °C) in artificial saliva (1700–0305, Pickering Laboratories, Mountain View, CA, USA; pH = 6.8) for up to 73 days to assess their simulated intra-oral durability. Disks were periodically removed, thoroughly rinsed in de-ionized (DI) water, and desiccated for subsequent analysis by XPS (as described before). Two disks with three XPS analysis spots were used per timepoint.

#### 2.2.4. Relative Amount of Surface Peptide

In order to compare the amount of KN3-LamLG3 and 2K-LamLG3 peptides on D-LamLG3 and S-LamLG3, respectively, disks were rinsed in phosphate-buffered saline (PBS) five times and 1% Triton X-100 after coating synthesis. Relative peptide concentration was then determined using a commercially available micro bicinchoninic acid kit (BCA; 23235, Thermo-Fisher, Waltham, MA, USA) by reacting the washed, desorbed disk in BCA solution A standard curve of both D-LamLG3 and S-LamLG3 was prepared and measured on a plate reader (Synergy HT, Biotek, Winooski, VT, USA) similar to past work [42,43]. Three disks per group were analyzed.

### 2.3. Oral Keratinocyte Response

Immortalized TERT-2/OKF-6 (BWH Cell Culture and Microscopy Core, Boston, MA, USA) oral keratinocytes from non-cancerous tissue from the floor of a human mouth were cultured in defined keratinocyte serum-free media (Gibco, Waltham, MA, USA) with 1% penicillin/streptomycin (Gibco) under standard cell culture conditions [44].

#### 2.3.1. Oral Keratinocyte Proliferation

Oral keratinocytes were seeded (6 × 10^4^), cultured for one day, and then washed in PBS and incubated for four hours in CCK8 solution (Dojindo, Kumamoto, Japan; 9:1 CCK8: oral keratinocyte media). Optical density (OD; λ = 450 nm) was obtained on a plate reader (Synergy HT, Biotek). OD values were blanked with virgin CCK8 solution similarly incubated (*n* = 8). The number of nuclei [based on 4′,6-diamidino-2-phenylindole dihydrochloride (DAPI; Sigma-Aldrich) staining as described below] was also quantified per field of view (FOV) to complement metabolic activity (3 FOVs per sample, *n* = 5).

#### 2.3.2. Lactase Dehydrogenase (LDH) Release

Initial cell viability was determined through lactate dehydrogenase (LDH) release. Cells were seeded as previously described. Disks were then transferred to a new wellplate. A CyQUANT colorimetric assay (C20300, Thermo-Fisher) was used to quantify the amount of LDH in solution, per the manufacturer’s instructions (*n* = 8).

#### 2.3.3. Immunofluorescence and Oral Keratinocyte Hemidesmosome Formation

Oral keratinocytes were seeded and cultured for one day as described and then fixed for ten minutes in 4% paraformaldehyde or ice-cold methanol (both Fisher Scientific, Waltham, MA, USA). Disks laden with cells were immersed in 5% bovine serum albumin (BSA) in PBS and then probed with a primary mouse monoclonal antibody (paraformaldehyde fixed) for integrin β4 [critical early marker for HD assembly [45]) NB10065599; Novus Biologicals, Littleton, CO, USA; 1:500] or primary rabbit polyclonal antibody (methanol fixed) for collagen XVII [(important late marker for HD assembly [46]) ab28440; Abcam, Cambridge, UK; 1:500] for 1 h at room temperature. Samples were immersed in an antimouse (A-11005; Invitrogen; 1:500) or antirabbit secondary (ab97037; abcam; 1:500) after extensive washing for 3 h. Samples were counterstained with DAPI. Total fluorescent intensity in each field was quantified after accounting for secondary-only controls at constant microscope settings across all samples (*n* = 5, 3 FOVs each). Micrographs (×10) Micrographs were obtained with an upright fluorescent microscope (DM 6B, Leica, Wetzlar, Germany) and analyzed in ImageJ (NIH, 2.0.0).

### 2.4. Statistical Analysis

Arithmetic mean values with one standard deviation on the mean are reported. Differences in mean between groups were assessed with a one-way analysis of variance (ANOVA) table followed by a Tukey’s HSD (honest significant difference) post hoc test. A *p* value of <0.05 was considered statistically significant. GraphPad Prism 8.3.0 (GraphPad Software) was used for statistical calculations. Figures were partially created with BioRender.

## 3. Results

### 3.1. Physical and Chemical Characterization of Peptide Coatings

**Figure 2.**
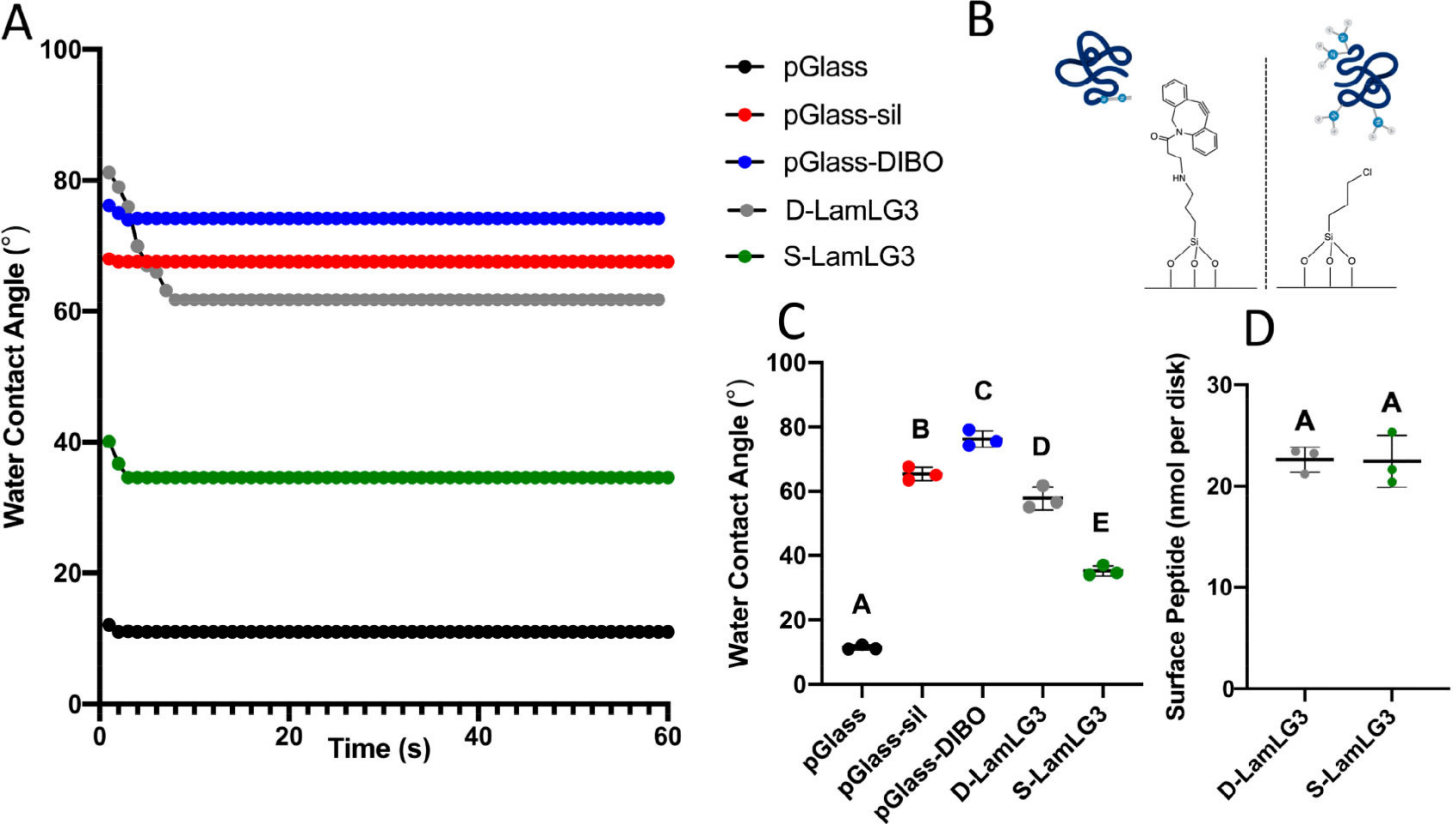
Dynamic water contact angles for LamLG3-coated and non-coated, control surfaces (**A**); schematic illustrating immobilization site(s) and chemistries of D-LamLG3 (**B**, left) and S-LamLG3 (**B**, right); water contact angles at equilibrium (**C**); and amount of peptide on D-LamLG3 and S-LamLG3 surfaces using a BCA assay (**D**). Differences in mean between groups were assessed with a one-way analysis of variance (ANOVA) table followed by a Tukey’s HSD (honest significant difference) post hoc test. A *p* value of <0.05 was considered statistically significant. Dissimilar letters denote statistically significant differences between groups.

**Figure 3.**
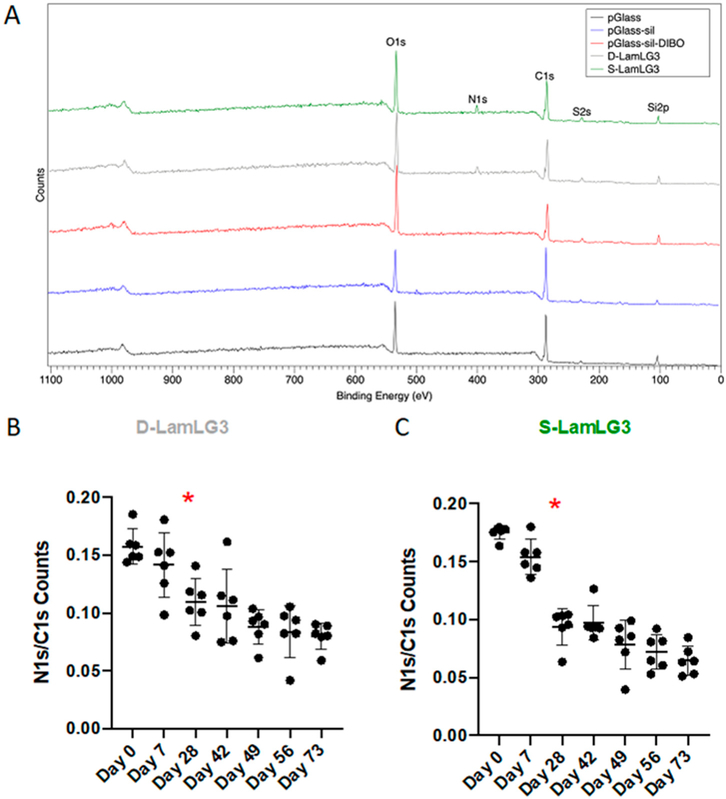
X-ray photoelectron spectroscopy spectra of peptide-coated (D-LamLG3, S-Lam-LG3) and non-coated, control surfaces. Full survey spectra of each coated and non-coated, control surface (**A**) and the ratio of N 1*s* X-ray photoelectron spectroscopy counts to C 1*s* counts (N 1*s*:C 1*s*) for D-LamLG3 (**B**) and S-LamLG3 (**C**) following up to 73 days in artificial saliva (37 °C). Differences in mean between groups were assessed with a one-way analysis of variance (ANOVA) table followed by a Tukey’s HSD (honest significant difference) post hoc test. A *p* value < 0.05 was considered statistically significant. The asterisk (*) denotes when the N 1*s*/C 1*s* signal was statistically significantly reduced compared to day 0.

Water contact angle measurements were performed (Figure 2A,C) after each iterative synthesis step for synthesizing surfaces immobilized with 2K-LamLG3 and KN3-LamLG3 peptides using silanization (S-LamLG3) and click chemistry (D-LamLG3). Marked differences were noted in the WCAs at equilibrium between S-LamLG3 (ca. 40°) and D-LamLG3 (ca. 60°) likely due to the differences in underlying chemistry (CPTES vs. CPTES and DIBO, respectively; shown in Figure 2B) or peptide conformation differences. The differences in peptide ability to restructure on the surface when in contact with water are also highlighted by the higher dynamic response (larger change in contact angle over time) for the D-LamLG3 surfaces compared to S-LamLG3 surfaces. In addition, a longer period to reach equilibrium in D-LamLG3 compared to S-LamLG3 was noted. The approximately 22 nmol per disk detected in both groups is similar to previous reports by other groups [47,48]. Despite these difference in WCAs, no differences were seen in the amount of peptide on the surfaces via a BCA assay (Figure 2D). XPS results (Figure 3A) further confirmed the formation of a LamLG3 coating through detection of Nitrogen (N 1*s* peak) from the amino acid backbone of the peptide on S-LamLG3 and D-LamLG3 surfaces. Silicon (Si 2*p*) was also present, likely from the underlying glass substrate [49].

### 3.2. Durability of Peptide Coatings

In order to determine the simulated oral cavity durability of our immobilized peptides, we incubated our coatings in simulated saliva for up to 73 days and then periodically performed XPS [Figure 3B (D-LamLG3) and Figure 3C (S-LamLG3)]. In both cases, the N 1*s*/C 1*s* signal significantly decreased following 28 days of incubation compared to day 0. However, this decrease in N 1*s*/C 1*s* signal was less pronounced in D-LamLG3 compared to S-LamLG3. No statistically significant differences were seen when comparing N 1*s*/C 1*s* between S-LamLG3 and D-LamLG3 at each timepoint (Figure S3). The underlying, more hydrophobic DIBO molecules than the CPTES molecules, and its associated hindrance of interactions with water molecules, are a plausible cause for the higher resistance to coating degradation of the D-LamLG3 surfaces. The CPTES molecules in S-LamLG3 surfaces are susceptible for hydrolysis and, thus, detachment from the surface [50]. Despite this, a notable peptide-associated signal remained after 73 days in both peptide-coated surfaces.

### 3.3. Keratinocytes Reponse on Peptide Coatings with Different Peptide Immobilization Chemistry

**Figure 4.**
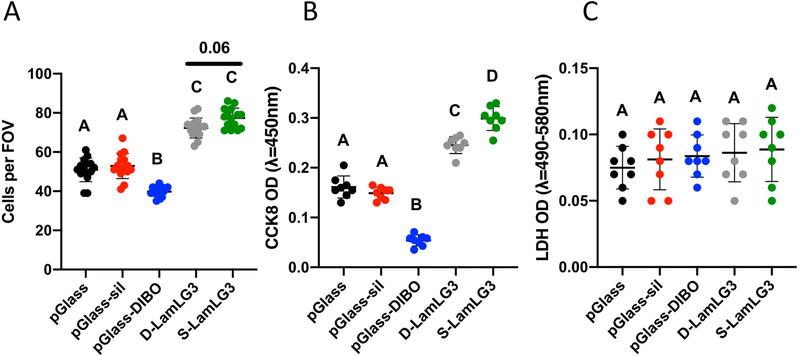
Oral keratinocyte proliferation [number of cells (**A**) and metabolic activity (**B**)] and cytotoxicity [LDH release (**C**)] of LamLG3-coated and non-coated, control surfaces after 1 day of culture. Differences in mean between groups were assessed with a one-way analysis of variance (ANOVA) table followed by a Tukey’s HSD (honest significant difference) post hoc test. A *p* value of <0.05 was considered statistically significant. Dissimilar letters denote statistically significant differences between groups.

**Figure 5.**
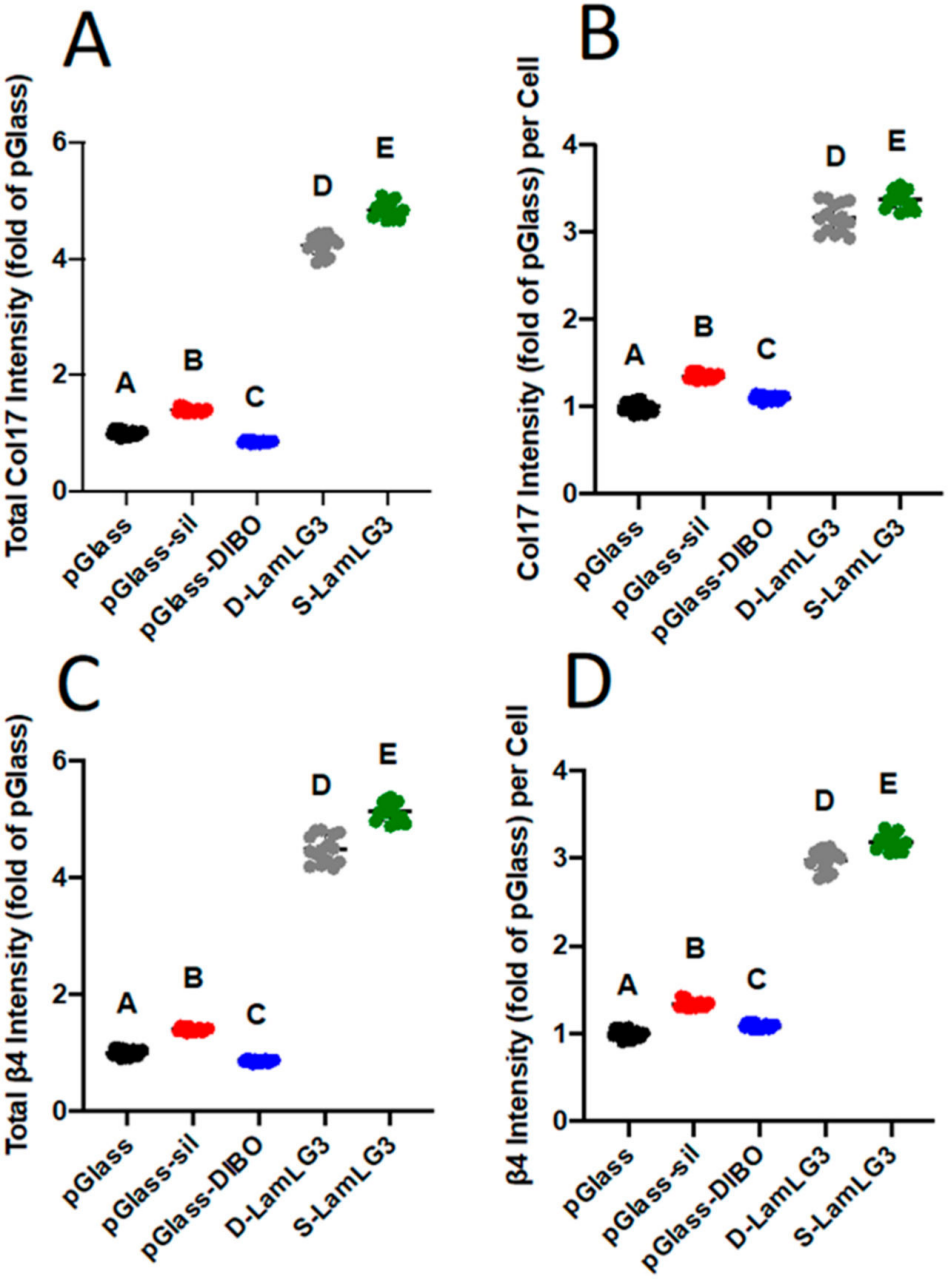
Oral keratinocyte hemidesmosome formation [total collagen XVII intensity (**A**), collagen XVII intensity normalized to number of cells (**B**), total integrin β4 intensity (**C**), and integrin β4 intensity normalized to number of cells (**D**)] of coated and non-coated, control surfaces at 1 day. Differences in mean between groups were assessed with a one-way analysis of variance (ANOVA) table followed by a Tukey’s HSD (honest significant difference) post hoc test. A *p* value of <0.05 was considered statistically significant. Dissimilar letters denote statistically significant differences between groups.

Oral keratinocytes were cultured on our coatings to determine the differences between S-LamLG3 and D-LamLG3 in promoting keratinocyte proliferation (Figure 4) and hemidesmosome formation (Figure 5). After one day of culture, proliferation was increased in terms of both increased numbers of cells (Figure 4A) and metabolic activity (Figure 4B) for both S-LamLG3 and D-LamLG3 compared to controls (pGlass, pGlass-sill, and pGlass-DIBO). No differences were seen in cytotoxicity (LDH release; Figure 4C). However, proliferation was nearly significant (*p* = 0.06; number of cells) or significantly (metabolic activity) increased on S-LamLG3 compared to D-LamLG3, suggesting that differences in immobilization methods affect resulting cellular behavior. An interesting observation was the marked reduction in keratinocyte proliferation on DIBO control surfaces compared to all other groups, which has not been previously reported.

Our targeted biological activity, hemidesmosome formation on dental implant surfaces (summarized in Figure 1), was finally evaluated. Semi-quantitative immunofluorescence was performed for both collagen XVII (Figure 5A,B; representative micrographs in Figure S4) and integrin β4 (Figure 5C,D; representative micrographs in Figure S5). Similar to proliferation, HD formation was significantly increased on both S-LamLG3 and D-LamLG3 compared to all controls. However, HD formation was significantly higher on S-LamLG3 compared to D-LamLG3, further emphasizing differences in immobilization methods affect resulting cellular behavior.

## 4. Discussion

Our specific aim was to compare the physiochemical properties and biological responses of LamLG3 peptide immobilized using silanization and click chemistry for improving dental implant outcomes through the upregulation of hemidesmosome formation in the soft tissue surrounding dental implants. The broader goal of this work was to use LamLG3 as a model peptide to study the effects of immobilization chemistry on cell adhesion peptide activity to engineer more efficacious coatings for biomedical devices. Our results show that the non-chemoselective route (silanization) resulted in increased keratinocyte proliferation and hemidesmosome formation compared to the chemoselective route (click chemistry).

Chemoselectivity of peptide immobilization for biological responses is an oft-cited design criteron to improve effectiveness for the desired biological activity with the selected peptides [51]. However, rationalization under the specific experimental conditions studied is rarely offered. Here, we show that this design criterion does not hold true under all conditions; indeed, here, the non-chemoselective route for immobilizing the LamLG3 peptides resulted in better biological outcomes (proliferation and HD formation). In some other systems, for example sensors [52] and antimicrobial surfaces [53], chemoselective immobilization results in better peptide activity. The ca. 20° difference in WCA between S-LamLG3 and D-LamLG3 and the longer time period for D-LamLG3 to reach equilibrium suggests that either underlying surfaces chemistry differences (presence of DIBO or not) and/or differences in number of residues where the peptide may be anchored (three for S-LamLG3 vs. one for D-LamLG3) may result in differences in peptide conformation, flexibility/mobility and/or orientation and subsequent cellular engagement. For example, recent work [54] has shown differences in osteogenic differentiation outcomes mediated by the identical osteogenic peptide immobilized through either amines or carboxyls on carbon nanotube surfaces. One potential cause for the differences we observed here is that KN3-LamLG3 peptides only provide one reactive site with the DIBO-modified surfaces, whereas 2K-LamLG3 peptides provide up to four (three free amines plus N-terminus) reactive sites with the CPTES-modified surfaces. As shown by dynamic WCAs, this can produce a more rigid molecular coating in the case of S-LamLG3 surfaces that might have facilitated appropriate cellular interactions. In spite of the speculative nature of this discussion, our results and results by others support that chemoselective peptide immobilization, in some but not all circumstances, can result in reduced peptide activity.

One design parameter for chemically immobilizing peptides is the spacer (or linker) design between the bioactive domain and the chemical domains necessary for anchorage. An ideal spacer maintains an independent structure and does not affect function of adjacent peptide domains; spacer type, length, and flexibility are generally tunable to achieve this goal [55]. While PEG spacers are very well studied for peptide immobilization, they require additional synthesis steps compared to amino acid-based spacers [10,56]. Previous research compared a classic amino acid spacer (GGG) and a novel spacer (GSGGG) with a “backbone bend” to separate an antimicrobial peptide domain from the surface. This novel GSGGG spacer showed enhanced antimicrobial activity compared to the classic spacer we used here [57,58]. Other work has compared a rigid spacer [(EAAAK)_4_] against a flexible spacer [(GGGGS)_4_]; the flexible spacer design showed more effective eukaryotic cell signaling but less effective antimicrobial activity [36,59]. Others have shown that flexibility per se is not the requisite design criteria but rather matching hydrophobicity/philicity of surrounding residues leads to maximal eukaryotic response [60]. While such general design principles are useful, fully optimized spacer design is likely system dependent.

Peptide orientation is another commonly cited design parameter when chemically immobilizing peptides. This is commonly modulated through changes in different chain positions, such as C-terminal, N-terminal and/or N-side-chain [10]. Our own work with the antimicrobial peptide GL13K has shown that orientation does not affect antimicrobial activity [61]. GL13K is antimicrobial both when immobilizedusingsilanizationandwhenrecombinantlyincorporatedintotheterminusofanelastin-like recombinamer [62]. On the other hand, LL37—another well-studied antimicrobial peptide for biomedical coatings—is sensitive to orientation [63]. Other work has shown that when an alpha-helical antimicrobial peptide is “standing” (helix *z*-axis perpendicular to surfaces), it interacts with bacterial cells faster than identical peptides “lying down” (helix *z*-axis parallel to surface) [64]. Similar orientation effects are well known for enzyme and the vastly explored RGD peptide immobilization [65,66]. Other factors such as the specific residue anchoring point (and number of them) affect activity as well, including peptide density [67,68]. Previous work has shown that peptide density, which we did not control here, can affect cell adhesion, differentiation, and focal adhesion formation [48,69,70]. Whether peptide density affects HD formation is unexplored to date. A number of strategies not discussed such as electric fields or chemical vapor deposition may also be viable for controlling peptide orientation [71–73]. On the whole, judicious selection of orientation is, again, likely system dependent. In particular for our D-LamLG3 system, future experiments are necessary to directly compare biological activity resulting from N - vs. C-terminus addition of the reactive azide.

An unexpected result of this work was the significantly reduced keratinocyte proliferation on pGlass-DIBO compared to all other groups; this result was reproducible under a number of different cell seeding schemes and we have seen this same trend with other cell types (unpublished observation). Others have reported the cytocompatibility of DIBO [74–76] under various conditions with various cells. Further work is necessary to discern this mechanism.

Overall, our results show that biological effects from cell adhesion peptides—LamLG3 in this case for dental implants—are dependent on the immobilization chemistry used. Additionally, the most effective immobilization chemistry for optimal peptide activity is heavily system dependent. Future work will include longer culture periods as HDs continue to form and mature and monitoring peptide degradation in human-isolated saliva with active enzymes. Despite the importance of early cell–peptide interactions dictating later cell fate, hemidesmosome formation does continue after one day of culture and later timepoints may provide additional data for better design of peptide coatings. Human saliva contains at least 1515 unique proteins [77], including enzymes, that made degrade immobilized peptides and render them less active or completely inactive. A more fundamental understanding of the many factors that go into cell adhesion peptide immobilization may result in faster translation of such proposed therapies through the reduction in variability and a better understanding of the entire system to enable industrial scale manufacturing.

## 5. Conclusions

We immobilized a cell adhesion peptide, LamLG3, on model surfaces to upregulate hemidesmosome formation by oral keratinocytes with the ultimate goal of reducing dental implant peri-implantitis. The non-chemoselective immobilization route, silanization, produced LamLG3 coatings that induced significantly higher proliferation and hemidesmosome formation in oral keratinocytes compared to LamLG3 coatings obtained using chemoselective copper-free click chemistry. Our results emphasize that the chemical route to immobilize peptides on biomedical surfaces has significant effects on cell adhesion and that chemoselectivity of the immobilization route is not always beneficial for enhancing the biological effects of the immobilized peptides. A deeper understanding of the effects of the selected chemical route of immobilization on biological activity, which has to be tested for the specific combination of peptide and targeted cellular response, is needed to develop more effective biomedical device peptide coatings

## Supplementary Material

SI **Supplementary Materials:** The following are available online at http://www.mdpi.com/2079-6412/10/6/560/s1, Figure S1: (A) HPLC analysis and (B) electrospray ionization (ESI) mass spectroscopy spectrum of S-LamLG3 (M_W_ = 2037.49 Da). Figure S2: (A) HPLC analysis and (B) ESI mass spectroscopy spectrum of D-LamLG3 (M_W_ = 1935.35 Da). Figure S3: Ratio of the N1s X-ray photoelectron spectroscopy to the C 1s counts (N 1s/C 1s) for D-LamLG3 vs. S-LamLG3 following up to 73 days in artificial saliva (37 °C). Differences in mean N 1s/C 1s counts between D-LamLG3 and S-LamLG3 were assessed with an unpaired t-test; there were no statistically significant differences at any timepoint (p > 0.05). Figure S4: Representative micrographs of oral keratinocyte Col17 immunofluorescence after one day of culture. The scale bar is 100 μm. Figure S5: Representative micrographs of oral keratinocyte integrin β4 immunofluorescence after one day of culture. The scale bar is 100 μm.

## References

[1] Collier JH; Segura T Evolving the use of peptides as components of biomaterials. Biomaterials 2011, 32, 4198–4204. 21515167 10.1016/j.biomaterials.2011.02.030 PMC3389831

[2] Hamley IW Small bioactive peptides for biomaterials design and therapeutics. Chem. Rev. 2017, 117, 14015–14041. 29227635 10.1021/acs.chemrev.7b00522

[3] Spicer CD; Pashuck ET; Stevens MM Achieving controlled biomolecule–biomaterial conjugation. Chem. Rev. 2018, 118, 7702–7743. 30040387 10.1021/acs.chemrev.8b00253 PMC6107854

[4] Mohamad NR; Marzuki NHC; Buang NA; Huyop F; Wahab RA An overview of technologies for immobilization of enzymes and surface analysis techniques for immobilized enzymes. Biotechnol. Biotechnol. Equip. 2015, 29, 205–220. 26019635 10.1080/13102818.2015.1008192 PMC4434042

[5] Koidou VP; Argyris PP; Skoe EP; Mota Siqueira J; Chen X; Zhang L; Hinrichs JE; Costalonga M; Aparicio C Peptide coatings enhance keratinocyte attachment towards improving the peri-implant mucosal seal. Biomater. Sci. 2018, 6, 1936–1945. 29850754 10.1039/c8bm00300a PMC6019193

[6] Chen X; Sevilla P; Aparicio C Surface biofunctionalization by covalent co-immobilization of oligopeptides. Colloids Surf. B Biointerfaces 2013, 107, 189–197. 23500730 10.1016/j.colsurfb.2013.02.005 PMC7473505

[7] Huettner N; Dargaville TR; Forget A Discovering cell-adhesion peptides in tissue engineering: Beyond RGD. Trends Biotechnol. 2018, 36, 372–383. 29422411 10.1016/j.tibtech.2018.01.008

[8] LeBaron RG; Athanasiou KA Extracellular matrix cell adhesion peptides: Functional applications in orthopedic materials. Tissue Eng. 2000, 6, 85–103. 10941205 10.1089/107632700320720

[9] Holmberg KV; Abdolhosseini M; Li Y; Chen X; Gorr SU; Aparicio C Bio-inspired stable antimicrobial peptide coatings for dental applications. Acta Biomater. 2013, 9, 8224–8231. 23791670 10.1016/j.actbio.2013.06.017 PMC3758876

[10] Costa F; Carvalho IF; Montelaro RC; Gomes P; Martins MCL Covalent immobilization of antimicrobial peptides (AMPs) onto biomaterial surfaces. Acta Biomater 2011, 7, 1431–1440. 21056701 10.1016/j.actbio.2010.11.005

[11] Mas-Moruno C; Su B; Dalby MJ Multifunctional coatings and nanotopographies: Toward cell instructive and antibacterial implants. Adv. Healthc. Mater. 2019, 8, 1801103. 10.1002/adhm.201801103 30468010

[12] Hoyos-Nogués M; Velasco F; Ginebra MP; Manero JM; Gil FJ; Mas-Moruno C Regenerating bone via multifunctional coatings: The blending of cell integration and bacterial inhibition properties on the surface of biomaterials. ACS Appl. Mater. Interfaces 2017, 9, 21618–21630. 28594999 10.1021/acsami.7b03127

[13] Hoyos-Nogués M; Buxadera-Palomero J; Ginebra MP; Manero JM; Gil FJ; Mas-Moruno C All-in-one trifunctional strategy: A cell adhesive, bacteriostatic and bactericidal coating for titanium implants. Colloids Surf. B Biointerfaces 2018, 169, 30–40. 29747028 10.1016/j.colsurfb.2018.04.050

[14] Hoyos-Nogués M; Brosel-Oliu S; Abramova N; Muñoz FX; Bratov A; Mas-Moruno C; Gil FJ Impedimetric antimicrobial peptide-based sensor for the early detection of periodontopathogenic bacteria. Biosens. Bioelectron. 2016, 86, 377–385. 27399935 10.1016/j.bios.2016.06.066

[15] Yu S; Zuo X; Shen T; Duan Y; Mao Z; Gao C A density gradient of VAPG peptides on a cell-resisting surface achieves selective adhesion and directional migration of smooth muscle cells over fibroblasts. Acta Biomater. 2018, 72, 70–81. 29635070 10.1016/j.actbio.2018.04.005

[16] Kam L; Shain W; Turner JN; Bizios R Selective adhesion of astrocytes to surfaces modified with immobilized peptides. Biomaterials 2002, 23, 511–515. 11761172 10.1016/s0142-9612(01)00133-8

[17] Brooks K; Yatvin J; McNitt CD; Reese RA; Jung C; Popik VV; Locklin J Multifunctional surface nanipulation using orthogonal click chemistry. Langmuir 2016, 32, 6600–6605. 27280689 10.1021/acs.langmuir.6b01591

[18] Sakala GP; Reches M Peptide-Based Approaches to Fight Biofouling. Adv. Mater. Interfaces 2018, 5, 1–26.

[19] Boskey AL; Villarreal-Ramirez E Intrinsically disordered proteins and biomineralization. Matrix Biol. 2016, 52–54, 43–59. 10.1016/j.matbio.2016.01.007 PMC4875856 26807759

[20] Gungormus M; Fong H; Kim IW; Evans JS; Tamerler C; Sarikaya M Regulation of in vitro calcium phosphate mineralization by combinatorially selected hydroxyapatite-binding peptides. Biomacromolecules 2008, 9, 966–973. 18271563 10.1021/bm701037x

[21] Sevilla P; Gil J; Aparicio C Relevant properties for immobilizing short peptides on biosurfaces. IRBM 2017, 38, 256–265.

[22] Chen X; Li Y; Aparicio C Biofunctional coatings for dental implants In Thin Films and Coatings in Biology; Springer: Berlin/Heidelberg, Germany, 2013; pp. 105–143. ISBN 978–94-007–2591-1.

[23] Shadish JA; DeForest CA Site-Selective Protein Modification: From Functionalized Proteins to Functional Biomaterials. Matter 2020, 2, 50–77.

[24] Saha B; Saikia J; Das G Correlating enzyme density, conformation and activity on nanoparticle surfaces in highly functional bio-nanocomposites. Analyst 2015, 140, 532–542. 25407103 10.1039/c4an01639d

[25] Sevilla P; Vining KV; Dotor J; Rodriguez D; Gil FJ; Aparicio C Surface immobilization and bioactivity of TGF-β1 inhibitor peptides for bone implant applications. J. Biomed. Mater. Res. Part B Appl. Biomater. 2016, 104, 385–394. 25826572 10.1002/jbm.b.33374

[26] Schwartz J; Avaltroni MJ; Danahy MP; Silverman BM; Hanson EL; Schwarzbauer JE; Midwood KS; Gawalt ES Cell attachment and spreading on metal implant materials. Mater. Sci. Eng. C 2003, 23, 395–400.

[27] Pagel M; Hassert R; John T; Braun K; Wießler M; Abel B; Beck-Sickinger AG Multifunctional coating improves cell adhesion on titanium by using cooperatively acting peptides. Angew. Chem. Int. Ed. Engl. 2016, 55, 4826–4830. 26938787 10.1002/anie.201511781

[28] Lummerstorfer T; Hoffmann H Click chemistry on surfaces: 1,3-dipolar cycloaddition reactions of azide-terminated monolayers on silica. J. Phys. Chem. B 2004, 108, 3963–3966.

[29] Buxadera-Palomero J; Calvo C; Torrent-Camarero S; Gil FJ; Mas-Moruno C; Canal C; Rodríguez D Biofunctional polyethylene glycol coatings on titanium: An in vitro-based comparison of functionalization methods. Colloids Surf. B Biointerfaces 2017, 152, 367–375. 28135680 10.1016/j.colsurfb.2017.01.042

[30] Godoy-Gallardo M; Mas-Moruno C; Yu K; Manero JM; Gil FJ; Kizhakkedathu JN; Rodriguez D Antibacterial properties of hLf1–11 peptide onto titanium surfaces: A comparison study between silanization and surface initiated polymerization. Biomacromolecules 2015, 16, 483–496. 25545728 10.1021/bm501528x

[31] Lee CT; Huang YW; Zhu L; Weltman R Prevalences of peri-implantitis and peri-implant mucositis: Systematic review and meta-analysis. J. Dent 2017, 62, 1–12. 28478213 10.1016/j.jdent.2017.04.011

[32] Overmann AL; Aparicio C; Richards JT; Mutreja I; Fischer NG; Wade SM; Potter BK; Davis TA; Bechtold JE; Forsberg JA; Orthopaedic osseointegration: Implantology and future directions. J. Orthop. Res. 2020, jor.24576. 10.1002/jor.24576 31876306

[33] O’Niel MB; Runge CL; Friedland DR; Kerschner JE Patient outcomes in magnet-based implantable auditory assist devices. JAMA Otolaryngol. Head Neck Surg. 2014, 140, 513–520. 24763485 10.1001/jamaoto.2014.484

[34] Atallah R; Leijendekkers RA; Hoogeboom TJ; Frölke JP Complications of bone-anchored prostheses for individuals with an extremity amputation: A systematic review. PLoS ONE 2018, 13, e0201821. 30092081 10.1371/journal.pone.0201821 PMC6084937

[35] Kim JM; Park WH; Min BM The PPFLMLLKGSTR motif in globular domain 3 of the human laminin-5 α3 chain is crucial for integrin α3β1 binding and cell adhesion. Exp. Cell Res. 2005, 304, 317–327. 15707596 10.1016/j.yexcr.2004.11.009

[36] Liu Z; Ma S; Lu X; Zhang T; Sun Y; Feng W; Zheng G; Sui L; Wu X; Zhang X; Reinforcement of epithelial sealing around titanium dental implants by chimeric peptides. Chem. Eng. J. 2019, 356, 117–129.

[37] Werner S; Huck O; Frisch B; Vautier D; Elkaim R; Voegel JC; Brunel G; Tenenbaum H The effect of microstructured surfaces and laminin-derived peptide coatings on soft tissue interactions with titanium dental implants. Biomaterials 2009, 30, 2291–2301. 19168216 10.1016/j.biomaterials.2009.01.004

[38] Atsuta I; Ayukawa Y; Kondo R; Oshiro W; Matsuura Y; Furuhashi A; Tsukiyama Y; Koyano K Soft tissue sealing around dental implants based on histological interpretation. J. Prosthodont. Res. 2016, 60, 3–11. 26725967 10.1016/j.jpor.2015.07.001

[39] Iorio V; Troughton LD; Hamill KJ Laminins: Roles and utility in wound repair. Adv. Wound Care 2015, 4, 250–263. 10.1089/wound.2014.0533 PMC4397997 25945287

[40] Kim E; Koo H Biomedical applications of copper-free click chemistry: In vitro, in vivo, and ex vivo. Chem. Sci 2019, 10, 7835–7851. 31762967 10.1039/c9sc03368h PMC6855312

[41] Meldal M; Tornøe CW Cu-Catalyzed Azide−Alkyne Cycloaddition. Chem. Rev. 2008, 108, 2952–3015. 18698735 10.1021/cr0783479

[42] Zhang N; Weir MD; Romberg E; Bai Y; Xu HHK Development of novel dental adhesive with double benefits of protein-repellent and antibacterial capabilities. Dent. Mater. 2015, 31, 845–854. 25990262 10.1016/j.dental.2015.04.013

[43] Zhang N; Chen C; Melo MA; Bai Y-X; Cheng L; Xu HH A novel protein-repellent dental composite containing 2-methacryloyloxyethyl phosphorylcholine. Int. J. Oral Sci. 2015, 7, 103–109. 25655010 10.1038/ijos.2014.77 PMC4817550

[44] Dickson MA; Hahn WC; Ino Y; Ronfard V; Wu JY; Weinberg RA; Louis DN; Li FP; Rheinwald JG Human keratinocytes that express hTERT and also bypass a p16INK4a-enforced mechanism that limits life span become immortal yet retain normal growth and differentiation characteristics. Mol. Cell. Biol. 2000, 20, 1436–1447. 10648628 10.1128/mcb.20.4.1436-1447.2000 PMC85304

[45] Dowling J; Yu QC; Fuchs E β4 Integrin is required for hemidesmosome formation, cell adhesion and cell survival. J. Cell Biol. 1996, 134, 559–572. 8707838 10.1083/jcb.134.2.559 PMC2120864

[46] Van den Bergh F; Eliason SL; Giudice GJ Type XVII collagen (BP180) can function as a cell−matrix adhesion molecule via binding to laminin 332. Matrix Biol. 2011, 30, 100–108. 21034821 10.1016/j.matbio.2010.10.005 PMC3057348

[47] Porté-Durrieu M; Guillemot F; Pallu S; Labrugère C; Brouillaud B; Bareille R; Amédée J; Barthe N; Dard M; Baquey C Cyclo-(DfKRG) peptide grafting onto Ti–6Al–4V: Physical characterization and interest towards human osteoprogenitor cells adhesion. Biomaterials 2004, 25, 4837–4846. 15120531 10.1016/j.biomaterials.2003.11.037

[48] Ma Y; Policastro GM; Li Q; Zheng J; Jacquet R; Landis WJ; Becker ML Concentration-Dependent hMSC Differentiation on Orthogonal Concentration Gradients of GRGDS and BMP-2 Peptides. Biomacromolecules 2016, 17, 1486–1495. 26959809 10.1021/acs.biomac.6b00088

[49] Sharma A; Jain H; Miller AC Surface modification of a silicate glass during XPS experiments. Surf. Interface Anal. 2001, 31, 369–374.

[50] Wasserman SR; Tao YT; Whitesides GM Structure and reactivity of alkylsiloxane monolayers formed by reaction of alkyltrichlorosilanes on silicon substrates. Langmuir 1989, 5, 1074–1087.

[51] Lempens EHM; Helms BA; Merkx M; Meijer EW Efficient and chemoselective surface immobilization of proteins by using aniline-catalyzed oxime chemistry. ChemBioChem 2009, 10, 658–662. 19241407 10.1002/cbic.200900028

[52] Gori A; Cretich M; Vanna R; Sola L; Gagni P; Bruni G; Liprino M; Gramatica F; Burastero S; Chiari M Multiple epitope presentation and surface density control enabled by chemoselective immobilization lead to enhanced performance in IgE-binding fingerprinting on peptide microarrays. Anal. Chim. Acta 2017, 983, 189–197. 28811026 10.1016/j.aca.2017.06.027

[53] Costa FMTA; Maia SR; Gomes PAC; Martins MCL Dhvar5 antimicrobial peptide (AMP) chemoselective covalent immobilization results on higher antiadherence effect than simple physical adsorption. Biomaterials 2015, 52, 531–538. 25818458 10.1016/j.biomaterials.2015.02.049

[54] Wang C; Cao G; Zhao T; Wang X; Niu X; Fan Y; Li X Terminal group modification of carbon nanotubes determines covalently bound osteogenic peptide performance. ACS Biomater. Sci. Eng. 2020, acsbiomaterials.9b01501. 10.1021/acsbiomaterials.9b01501 33464866

[55] Argos P An investigation of oligopeptides linking domains in protein tertiary structures and possible candidates for general gene fusion. J. Mol. Biol. 1990, 211, 943–958. 2313701 10.1016/0022-2836(90)90085-Z

[56] Nie B; Long T; Li H; Wang X; Yue B A comparative analysis of antibacterial properties and inflammatory responses for the KR-12 peptide on titanium and PEGylated titanium surfaces. RSC Adv. 2017, 7, 34321–34330.

[57] Wisdom C; VanOosten SK; Boone KW; Khvostenko D; Arnold PM; Snead ML; Tamerler C Controlling the biomimetic implant interface: Modulating antimicrobial activity by spacer design. J. Mol. Eng. Mater. 2016, 04, 1640005. 10.1142/S2251237316400050 PMC5604879 28936427

[58] Wisdom EC; Zhou Y; Chen C; Tamerler C; Snead ML Mitigation of Peri-implantitis by Rational Design of Bifunctional Peptides with Antimicrobial Properties. ACS Biomater. Sci. Eng. 2019, 6. 10.1021/acsbiomaterials.9b01213 PMC7255409 32467858

[59] Liu Z; Ma S; Duan S; Xuliang D; Sun Y; Zhang X; Xu X; Guan B; Wang C; Hu M; Modification of titanium substrates with chimeric peptides comprising antimicrobial and titanium-binding motifs connected by linkers to inhibit biofilm formation. ACS Appl. Mater. Interfaces 2016, 8, 5124–5136. 26863404 10.1021/acsami.5b11949

[60] Craig JA; Rexeisen EL; Mardilovich A; Shroff K; Kokkoli E Effect of linker and spacer on the design of a fibronectin-mimetic peptide evaluated via cell studies and AFM adhesion forces. Langmuir 2008, 24, 10282–10292. 18693703 10.1021/la702434p

[61] Lan C A Multi-Functional st-ELR Scaffold for Dentin Regeneration; University of Minnesota: Minneapolis, MN, USA, 2016.

[62] Acosta S; Quintanilla L; Alonso M; Aparicio C; Rodríguez-Cabello JC Recombinant AMP/Polypeptide Self-Assembled Monolayers with Synergistic Antimicrobial Properties for Bacterial Strains of Medical Relevance. ACS Biomater. Sci. Eng. 2019, 5, 4708–4716. 10.1021/acsbiomaterials.9b00247 33448843

[63] Gabriel M; Nazmi K; Veerman EC; Amerongen AVN; Zentner A Preparation of LL-37-grafted titanium surfaces with bactericidal activity. Bioconjug. Chem 2006, 17, 548–550. 16536489 10.1021/bc050091v

[64] Li Y; Wei S; Wu J; Jasensky J; Xi C; Li H; Xu Y; Wang Q; Marsh ENG; Brooks CL; Effects of peptide immobilization sites on the structure and activity of surface tethered antimicrobial peptides. J. Phys. Chem. C 2015, 119, 7146–7155.

[65] Schroeder MM; Wang Q; Badieyan S; Chen Z; Marsh ENG Effect of surface crowding and surface hydrophilicity on the activity, stability and molecular orientation of a covalently tethered enzyme. Langmuir 2017, 33, 7152–7159. 28654290 10.1021/acs.langmuir.7b00646

[66] Vida Y; Collado D; Najera F; Claros S; Becerra J; Andrades JA; Perez-Inestrosa E Dendrimer surface orientation of the RGD peptide affects mesenchymal stem cell adhesion. RSC Adv. 2016, 6, 49839–49844.

[67] Li Y; Ogorzalek TL; Wei S; Zhang X; Yang P; Jasensky J; Brooks CL; Marsh ENG; Chen Z Effect of immobilization site on the orientation and activity of surface-tethered enzymes. Phys. Chem. Chem. Phys. 2018, 20, 1021–1029. 29235592 10.1039/c7cp06063g

[68] Zou X; Wei S; Badieyan S; Schroeder M; Jasensky J; Brooks CL; Marsh ENG; Chen Z Investigating the effect of two-point surface attachment on enzyme stability and activity. J. Am. Chem. Soc. 2018, 140, 16560–16569. 30403342 10.1021/jacs.8b08138

[69] Koo LY; Irvine DJ; Mayes AM; Lauffenburger DA; Griffith LG Co-regulation of cell adhesion by nanoscale RGD organization and mechanical stimulus. J. Cell Sci. 2002, 115, 1423–1433. 11896190 10.1242/jcs.115.7.1423

[70] Moore NM; Lin NJ; Gallant ND; Becker ML Synergistic enhancement of human bone marrow stromal cell proliferation and osteogenic differentiation on BMP-2-derived and RGD peptide concentration gradients. Acta Biomater. 2011, 7, 2091–2100. 21272672 10.1016/j.actbio.2011.01.019

[71] Martin LJ; Akhavan B; Bilek MMM Electric fields control the orientation of peptides irreversibly immobilized on radical-functionalized surfaces. Nat. Commun. 2018, 9, 357. 29367659 10.1038/s41467-017-02545-6 PMC5783936

[72] Youn YH; Lee SJ; Choi GR; Lee HR; Lee D; Heo DN; Kim BS; Bang JB; Hwang YS; Correlo VM; Simple and facile preparation of recombinant human bone morphogenetic protein-2 immobilized titanium implant via initiated chemical vapor deposition technique to promote osteogenesis for bone tissue engineering application. Mater. Sci. Eng. C 2019, 100, 949–958. 10.1016/j.msec.2019.03.048 30948131

[73] Song IT; Lee M; Lee H; Han J; Jang JH; Lee MS; Koh GY; Lee H PEGylation and HAylation via catechol: α-Amine-specific reaction at N-terminus of peptides and proteins. Acta Biomater. 2016, 43, 50–60. 27424082 10.1016/j.actbio.2016.07.018

[74] Lee HJ; Fernandes-Cunha GM; Putra I; Koh W-G; Myung D Tethering Growth Factors to Collagen Surfaces Using Copper-Free Click Chemistry: Surface Characterization and in Vitro Biological Response. ACS Appl. Mater. Interfaces 2017, 9, 23389–23399. 28598594 10.1021/acsami.7b05262

[75] Lee HJ; Fernandes-Cunha GM; Na K-S; Hull SM; Myung D Bio-Orthogonally Crosslinked, In Situ Forming Corneal Stromal Tissue Substitute. Adv. Healthc. Mater. 2018, 7, 1800560. 10.1002/adhm.201800560 PMC6417806 30106514

[76] Ashley GW; Henise J; Reid R; Santi DV Hydrogel drug delivery system with predictable and tunable drug release and degradation rates. Proc. Natl. Acad. Sci. USA 2013, 110, 2318–2323. 23345437 10.1073/pnas.1215498110 PMC3568318

[77] Schweigel H; Wicht M; Schwendicke F Salivary and pellicle proteome: A datamining analysis. Sci. Rep. 2016, 6, 1–12. 27966577 10.1038/srep38882 PMC5155218

