# Supplementary material for: Surface Immobilization Chemistry of a Laminin-Derived Peptide Affects Keratinocyte Activity
Source: Coatings (Basel). Author manuscript; Available in PMC 2020 Aug 26. (PMC7448695; doi:10.3390/coatings10060560)
Supplement: SI — Supplementary Materials: The following are available online at http://www.mdpi.com/2079-6412/10/6/560/s1, Figure S1: (A) HPLC analysis and (B) electrospray ionization (ESI) mass spectroscopy spectrum of S-LamLG3 (MW = 2037.49 Da). Figure S2: (A) HPLC analysis and (B) ESI mass spectroscopy spectrum of D-LamLG3 (MW = 1935.35 Da). Figure S3: Ratio of the N1s X-ray photoelectron spectroscopy to the C 1s counts (N 1s/C 1s) for D-LamLG3 vs. S-LamLG3 following up to 73 days in artificial saliva (37 °C). Differences in mean N 1s/C 1s counts between D-LamLG3 and S-LamLG3 were assessed with an unpaired t-test; there were no statistically significant differences at any timepoint (p > 0.05). Figure S4: Representative micrographs of oral keratinocyte Col17 immunofluorescence after one day of culture. The scale bar is 100 μm. Figure S5: Representative micrographs of oral keratinocyte integrin β4 immunofluorescence after one day of culture. The scale bar is 100 μm. [file NIHMS1615748-supplement-SI.docx]

**Supplemental Files**

Surface immobilization chemistry of a laminin-derived peptide affects keratinocyte activity

Nicholas G. Fischer ^1^, Jiahe He^1^ and Conrado Aparicio ^1,^*

**^1^** Minnesota Dental Research Center for Biomaterials and Biomechanics, University of Minnesota, 515 Delaware Street S.E., Minneapolis, MN, USA; [fisc0456@umn.edu](mailto:fisc0456@umn.edu) (N.G.F.) and [he000086@umn.edu](mailto:he000086@umn.edu) (J.H.)

***** Correspondence: [apari003@umn.edu](mailto:apari003@umn.edu) (C.A.)

**A)**

**
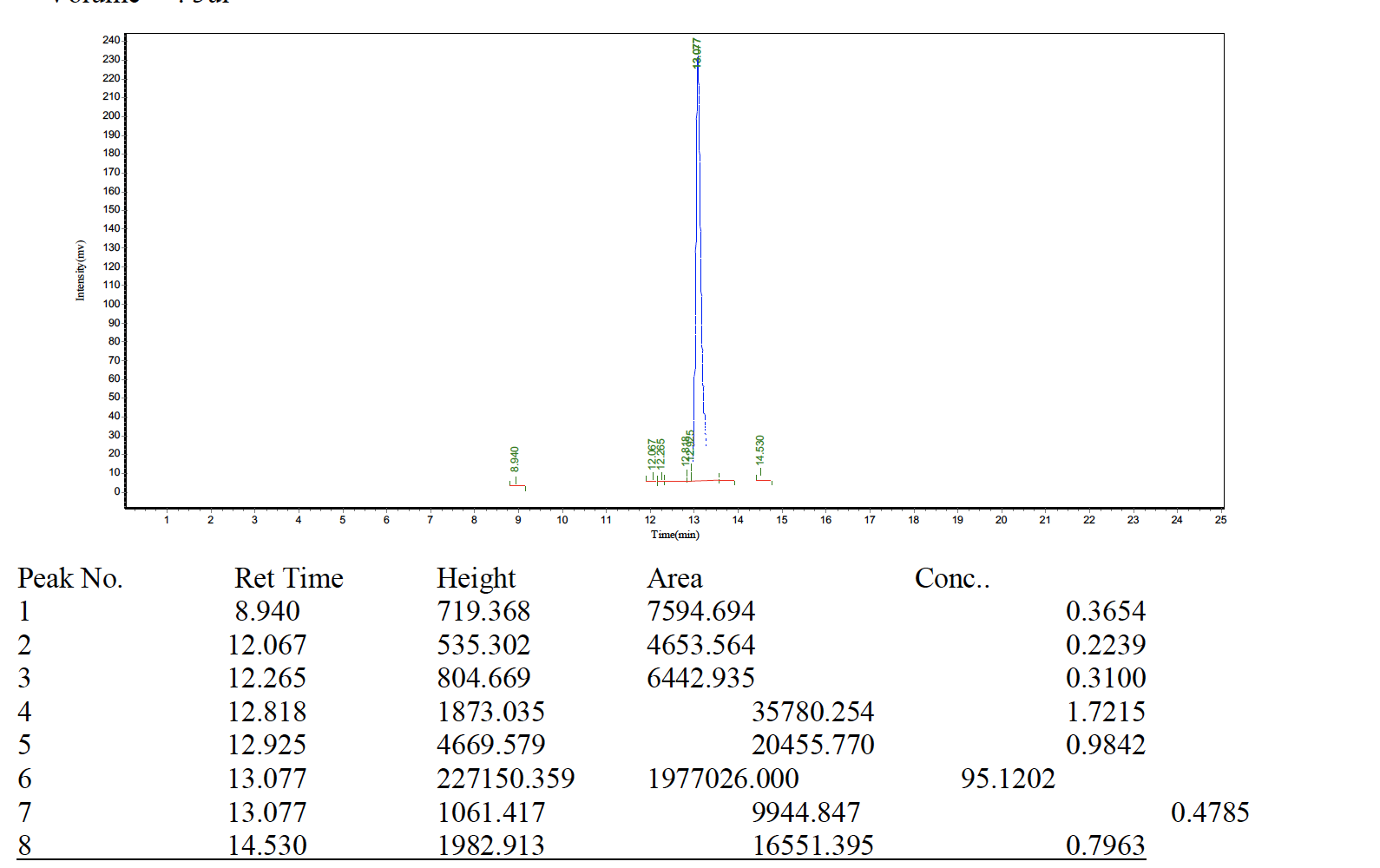
**

**B)**


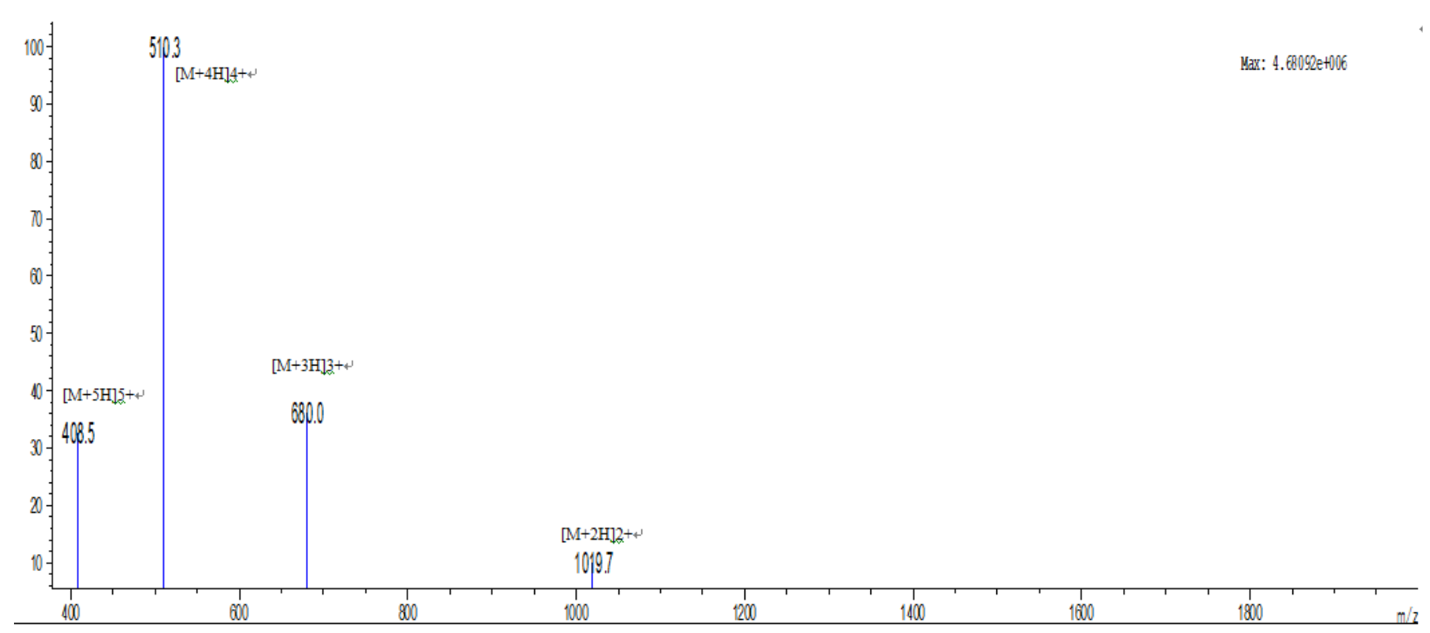


**Figure S1**. A) HPLC analysis and B) mass spectroscopy spectrum of S-LamLG3 (MW=2037.49 Da).

**A)**


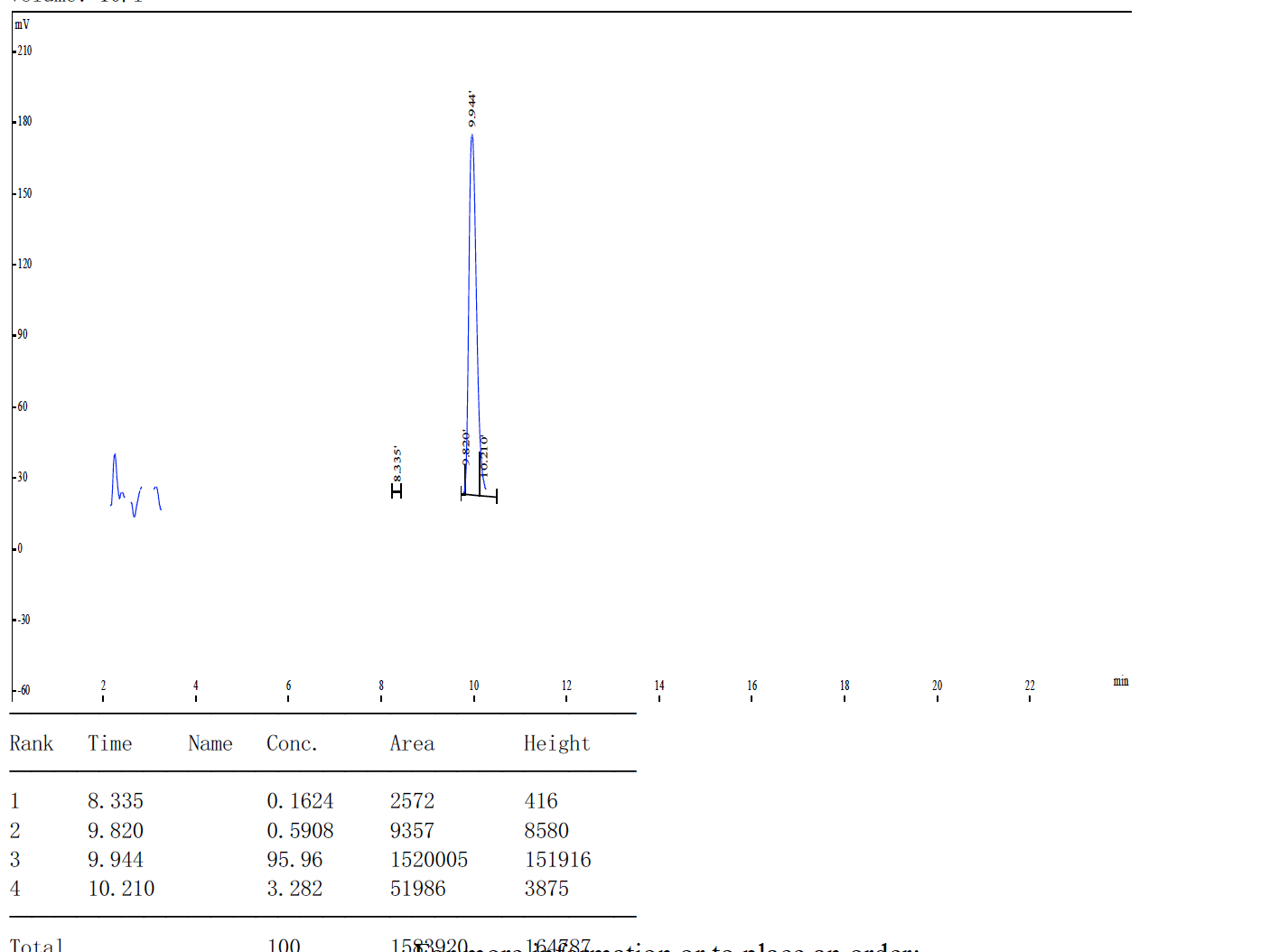


**B)**


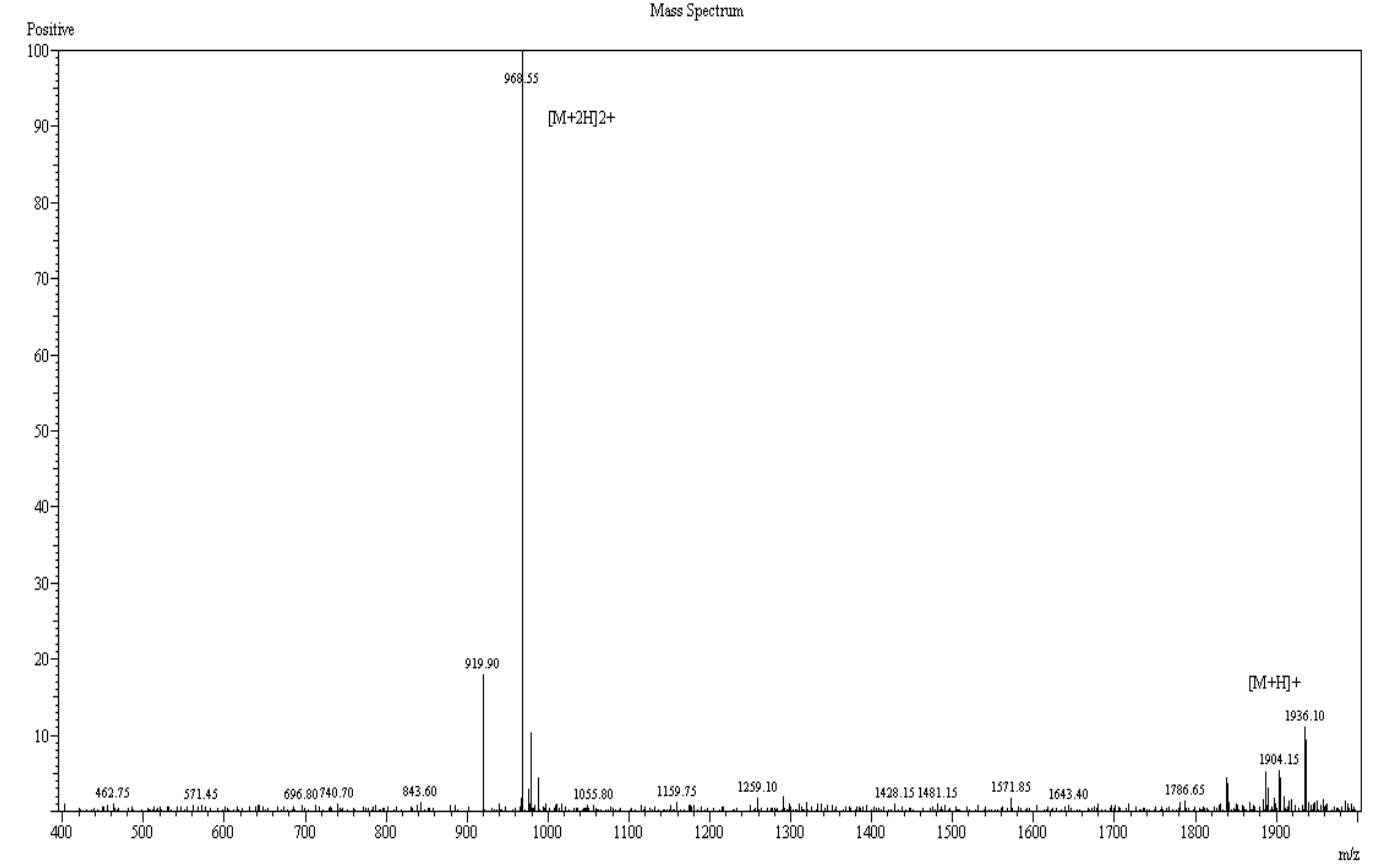


**Figure S2**. A) HPLC analysis and B) mass spectroscopy spectrum of D-LamLG3 (MW=1935.35 Da)

**Figure S3.** Representative micrographs of oral keratinocyte Col17 immunofluorescence after one day of culture. The scale bar is 100 μm.

**Figure S4.** Representative micrographs of oral keratinocyte integrin β4 immunofluorescence after one day of culture. The scale bar is 100 μm.

**Figure S5.** Ratio of the N1s X-ray photoelectron spectroscopy to the C1s counts (N1s/C1s) for D-LamLG3 vs. S-LamLG3 following up to 73 days in artificial saliva (37°C). Differences in mean N1s/C1s Counts between D-LamLG3 and S-LamLG3 were assessed with an unpaired *t-*test; there were no statistically significant differences at any time point (*p* >0.05).
